# Functional Development of Preterm Children Born from Singleton and Multiple Pregnancies: Preliminary Study

**DOI:** 10.34763/jmotherandchild.20212501.d-20-00002

**Published:** 2021-10-11

**Authors:** Aneta M Suder, Agnieszka J Gniadek, Agnieszka M Micek, Dorota W Pawlik

**Affiliations:** 1 Clinical Department of Neonatology, University Hospital in Cracow, postgraduate student at the Faculty of Health Sciences, Jagiellonian University Medical College Cracow Poland; 2 Institute of Nursing and Midwifery, Faculty of Health Sciences, Jagiellonian University Medical College Cracow Poland; 3 Clinical Department of Neonatology, University Hospital in Cracow Cracow Poland

**Keywords:** preterm children, functional development, Munich Functional Developmental Diagnostics

## Abstract

**Background:**

In recent years an increasing number of multiple pregnancies have been observed, which is a result of advancements made in reproductive technologies for infertility treatments as well as a tendency for women to delay childbearing until later in life. The risk of preterm birth in the case of a twin pregnancy is five to seven times higher than that of a singleton pregnancy, and in the case of triplet pregnancy, the risk is even 10 times higher. The objective of the study was to assess and compare the functional development of children aged between 2 and 2.5 who were prematurely born from singleton, twin and triplet pregnancies.

**Material and methods:**

The study was carried out in a group of 43 children aged between 2 and 2.5 who were born prematurely (between the 32nd and 36th week of pregnancy) in 2017 and 2018. Group I was made up of 10 children born from singleton pregnancies, group II included 12 children born from six twin pregnancies and group III consisted of 21 children born from seven triplet pregnancies. The evaluation of functional development was conducted using the Munich Functional Developmental Diagnostics.

**Results:**

There were no statistically significant differences in functional development between the studied singletons, twins and triplets. In the examined groups of singletons, twins and triplets, the calculated quotient medians for the 50th percentile approximated 1, which means that development was typical and did not differ from the development of the general population. In turn, for the 95th percentile, the median scores usually approximated 0.8, which also indicated that there was no significant delay in development. Had scores been higher than 1, this might have indicated a delay.

**Conclusions:**

On the basis of the study group, no relationship was found between the multiplicity of pregnancies and the functional development of premature babies born between the 32nd and 36th weeks of gestation.

## Introduction

In recent years an increasing number of multiple pregnancies has been observed, which is a result of advancement in reproductive technologies for infertility treatment as well as a tendency for women to delay childbearing until later in life. Multifoetal pregnancies are considered to be high-risk pregnancies because of a higher frequency of complications, such as preterm birth, intrauterine growth restrictions, polyhydramnios, oligohydramnioss or malposition of foetus. The risk of preterm birth in the case of twin pregnancy is five to seven times as high as in the case of a singleton pregnancy, and in the case of triplet pregnancy, it is even ten times higher [1,2,3]. About 50% of twins and over 90% of triplets are born before the 37th week of pregnancy or with a birth weight lower than 2500 g [1]. The aetiology of preterm birth in the case of multiple pregnancies is probably multicausal. The perinatal mortality rate is three times higher in the case of twin pregnancies in comparison with singleton pregnancies [4]. Survival of preterm infants born from multiple pregnancies is affected by numerous factors, including the frequency and type of complications in pregnancy, type and history of multiple pregnancies, birth weight, inequality in foetal growth, gender, the mother’s concomitant diseases and the mother’s race [1]. Although multiple pregnancies constitute only about 3% of all live births, they impose a three times greater financial burden on health care systems in comparison with singleton pregnancies because of a more frequent need for caesarean section and a higher likelihood of multiple preterm infants being admitted to neonatal hospital wards [5]. According to the Statisctic Polish, in 2018, 9,964 live births were from multiple pregnancies, including 9,706 twins and 238 triplets [6]. The World Health Organization (WHO) defines preterm babies as babies born after the 22nd week of pregnancy and before the 37th week is completed (before the 259th day since the first day of the last menstruation) regardless of the birth weight [7]. Taking into account the week of pregnancy and, consequently, the extent of physiological and metabolic maturity, preterm babies can be divided into the following groups [8,9]:

- extremely preterm: born before the 28th week of pregnancy
- very preterm: born between the 28th (0/7) and 31st (6/7) week of pregnancy
- moderate preterm: born between the 32nd (0/7) and 33rd (6/7) week of pregnancy
- late preterm: born between the 34th (0/7) and 36th (6/7) week of pregnancy

From birth, premature babies require special interdisciplinary care from numerous specialists, including a neonatologist, neurologist, ophthalmologist, physiotherapist, speech therapist, or psychologist. Early psychomotor diagnosis and regular medical check-ups are a chance for quick treatment of disorders and delays in particular spheres of premature children’s development.

Moderate and late premature babies have a greater risk of developing neurodevelopmental disorders, mainly in the cognitive sphere, than do their term-born peers [10]. The current studies do not show any unambiguous conclusions about the psychomotor development of premature babies born as singles, twins or triplets.

### Objective

The objective of the study was to assess and compare the functional development of children aged between 2 and 2.5 who were prematurely born from singleton, twin and triplet pregnancies.

## Material and methods

The study was carried out in a group of 43 children aged between 2 and 2.5 (calendar age) who were born prematurely between the 32nd and 36th week of pregnancy without severe neurological disorders. Group I was made up of ten children born from singleton pregnancies, group II included six pairs of twins and group III consisted of 21 triplets coming from seven families. The three groups of preterm babies are comparable in terms of maturity during labour.

Inclusion criteria:

- Children aged between 2 and 2.5 at the time of the study who were born prematurely in 2017 and 2018 between the 32nd and 36th week of pregnancy and hospitalised in the Clinical Department of Neonatology of the University Hospital in Krakow.
- Children with no neurological damage, severe sight and hearing disorders or severe bronchopulmonary dysplasia.
- Both parents’ consent for their children’s participation in the study.

Exclusion criteria:

- Preterm children born between the 32nd and 36th week of pregnancy with grade III and IV intraventricular haemorrhage, severe bronchopulmonary dysplasia or severe neurological damage (e.g., periventricular leukomalacia, birth defects of the brain and spinal cord).
- Preterm children suffering from severe sight or hearing disorders.
- Lack of consent for children’s participation in the study from at least one parent.

Exclusion criteria during the diagnosis:

- Children’s unwillingness to cooperate with the researcher.
- Children’s failure to complete all tasks within 50 minutes.
- Parents’ resignation from their children’s participation in the study.

The study was carried out in 2018 and 2019 in an outpatient clinic of the Clinical Department of Neonatology of the University Hospital at 23 Kopernika Street in Krakow with the approval of the hospital authorities and the Bioethics Committee (nr 1072.6120.20.2019). The examination of the children was carried out by one person who underwent training and received a certificate entitling him to perform a diagnosis according to the Munich Functional Developmental Diagnostics. Medical records were analysed during the study in order to obtain information about birth parameters of the examined children, such as birth weight, body length, head circumference, chest circumference and Apgar score. In order to assess functional development, Munich Functional Developmental Diagnostics for the second and third year of life were used, which allowed for a quantitative description of possible delays in seven spheres (motor development, manual functions, scope of perception, speech development, speech understanding, social development and the scope of independence).

Two scores were established for each child. The final variables determining children’s functional development in each of the seven spheres were calculated by dividing the number expressing child’s biological age by the number defining the standard age at which 50% and 95% of children reach the same level of functioning within the given sphere as the examined child. Therefore, the score >1 for the standard of the 50th percentile means that the examined child reaches the particular level of development later than 50% of the total population of children aged between 2 and 3, whereas the score >1 for the standard of the 95th percentile means that the child reaches this particular level later than 95% of the population of children aged between 2 and 3.^11^

### Statistical analysis

Categorical variables were described by counts and percentages. Due to a relatively low number of subjects in the groups, the normality of quantitative variables was assessed visually by examining the histograms and by means of a formal Shapiro-Wilk test. The normally distributed data were expressed as means with standard deviation (SD) and the non-normally distributed data as quartiles. The differences between quantitative variables were assessed using the Chi-square test of independence or Fisher exact test. The significance of differences in functional development in the seven examined spheres between singleton, twin and triplet pregnancies was assessed by means of a nonparametric Kruskal Wallis ANOVA rank test. All the analyses were conducted with the application of statistical software R 3.6.1 version. (R Core Team (2017). R: A language and environment for statistical computing. R Foundation for Statistical Computing, Vienna, Austria. URL https://www.R-project.org/) In this study the level of significance was set as α = 0.05.

## Results

Group I was made up of ten children (23%) born from singleton pregnancies, group II included 12 children (28%) born from twin pregnancies and group III consisted of 21 children (49%) born from triplet pregnancies. All these children were born by caesarean section. The examined group consisted of 18 boys (42%) and 25 girls (58%). Birth parameters of the examined group were congruent with normal distribution as far as anthropometric characteristics were concerned; however, the assessment of newborns’ conditions measured on the Apgar scale in the first and fifth minute of life indicated significant deviations from the normal distribution (Table 1.)

**Table 1:** Birth parameters of the examined group

Variable	Group			p^*^
	1 (N = 10)	2 (N = 12)	3 (N = 21)	
**Weight [g], mean (SD)**	1919 (481.33)	2004.17 (256.67)	1669.38 (279.38)	0.017
**Body length [cm], mean (SD)**	45.9 (3.57)	47.25 (3.55)	43.38 (3.47)	0.011
**Head circumference [cm], mean (SD)**	30.5 (2.32)	31.08 (1.73)	30.14 (1.68)	0.384
**Chest circumference [cm], mean (SD)**	27 (2.36)	27.08 (1.73)	26.14 (2.15)	0.376
**Apgar scale 1’, median (Q**_1_ **–Q**_3_ **)**	8 (6.5-9.75)	7.5 (7-10)	7 (6-8)	0.374
**Apgar scale 5’, median (Q**_1_ **–Q**_3_ **)**	8.5 (8-9.75)	9 (7-10)	8 (7-8)	0.052

SD, standard deviation. Q1, Q3 – lower and upper quartile. *p value based on ANOVA or Kruskal Wallis test depending on normality of distribution. Apgar scale parameters are presented by quartiles due to skewness of distribution; comparison between groups was performed using the Kruskal Wallis test.

Socioeconomic factors play a role in shaping the development trajectories of premature babies. The study group included such factors as gender, place of birth, parents’ education, economic status of the family, number of siblings and whether the family is full or mono-parent. Statistically significant differences between the studied groups were shown only in the number of siblings. (Table 2.)

**Table 2:** Characteristics of the study group

Variable n (%)	Category	Group 1 (N=10)	Group 2 (N=12)	Group 3 (N=21)	p#
**sex**	**girl** **boy**	4 (40) 6 (60)	4 (33.33) 8 (66.67)	10 (47.62) 11 (52.38)	0.719
**place of residence**	**village** **city up to 100** **city with over 100**	4 (40) 2 (20) 4 (40)	4 (33.33) 4 (33.33) 4 (33.33)	9 (42.86) 6 (28.57) 6 (28.57)	0.938
**mother’s education**	**basic** **medium** **higher**	2 (20) 3 (30) 5 (50)	0 (0) 2 (16.67) 10 (83.33)	0 (0) 9 (42.86) 12 (57.14)	0.052
**father’s education**	**basic** **medium** **higher**	2 (20) 4 (40) 4 (40)	0 (0) 4 (33.33) 8 (66.67)	0 (0) 6 (28.57) 15 (71.,43)	0.092
**economic situation**	**weak** **sufficient** **very good**	1 (10) 8 (80) 1 (10)	0 (0) 6 (50) 6 (50)	3 (14.29) 9 (42.86) 9 (42.86)	0.187
**family status**	**single parent** **full family**	0 (0) 10 (100)	2 (16.67) 10 (83.33)	0 (0) 21 (100)	0.067
**number of siblings**	0 1 2 3	5 (50) 2 (20) 3 (30) 0 (0)	0 (0) 10 (83.33) 0 (0) 2 (16.67)	0 (0) 0 (0) 15 (71.43) 6 (28.57)	0.000

#based on chi square test or Fisher exact test

No statistically significant differences were observed between the examined singletons, twins and triplets in any of the examined seven spheres of development including walking skills, manual functions, perception, speech development and understanding, scope of independence, or social development (Table 3). It was proven in the analyses of the quotient values that the examined preterm children born between the 32nd and 36th week of pregnancy and suffering from no neurological disorders do not significantly differ from the average population of full-term children as far as the analyzed seven spheres of development are concerned. In the examined groups of singletons, twins and triplets, the calculated quotient medians for the 50th percentile approximated 1, which means that their development is typical and does not differ from the development of the general population. In addition, for the 95th percentile, median scores usually approximated 0.8, which also meant there was no significant delay in children’s development, which might be the case if the score was higher than 1. The lowest quotient values both for the 50th and 95th percentile were observed in manual skilfulness and then in walking age, which might mean that in the case of these functions development of preterm children is the most similar to the development of the general population of children of this age. The highest quotient values were observed in the case of the perception age, speaking age, speech understanding and social development. Analogically, in these spheres preterm children’s scores were slightly less congruent with the scores of the general population (Table 3).

**Table 3:** The results of the Munich Functional Developmental Diagnostics for singletons, twins and triplets

Sphere of development	pregnancy	n	q2 (q1-q3) 50%^#^	p*	q2 (q1-q3) 95%^##^	p*
**Walking age**	singleton	10	1.08 (0.99-1.13)	0.24	0.83 (0.81-0.92)	0.28
	twin	12	1.05 (1.01-1.08)		0.85 (0.83-0.88)	
	triplet	21	1.01 (0.96-1.08)		0.81 (0.77-0.84)	
**Manual skillfulness age**	singleton	10	1.02 (0.94-1.13)	0.67	0.76 (0.7-0.85)	0.54
	twin	12	1 (0.94-1.04)		0.76 (0.72-0.76)	
	triplet	21	1.01 (0.93-1.04)		0.75 (0.69-0.76)	
**Perception age**	singleton	10	1.11 (1.07-1.15)	0.26	0.89 (0.84-0.92)	0.14
	twin	12	1.1 (1.08-1.12)		0.87 (0.82- 0.91)	
	triplet	21	1.04 (1-1.13)		0.81 (0.79-0.91)	
**Active speech age**	singleton	10	1.23 (1.16-1.31)	0.78	0.88 (0.83-0.97)	0.69
	twin	12	1.15 (1.09-1.24)		0.81 (0.79-0.91)	
	triplet	21	1.18 (1.08-1.3)		0.84 (0.81-0.89)	
**Speech understanding age**	singleton	10	1.1 (1.04-1.14)	0.62	0.86 (0.82-0.88)	0.91
	twin	12	1.13 (1.05-1.19)		0.84 (0.81-0.88)	
	triplet	21	1.12 (1.08-1.3)		0.86 (0.81-0.98)	
**Social development age**	singleton	10	1.15 (1.03-1.28)	0.82	0.81 (0.76-1)	0.81
	twin	12	1.11 (1.08-1.26)		0.88 (0.8-0.98)	
	triplet	21	1.13 (1.1-1.3)		0.96 (0.81-0.96)	
**Independence age**	singleton	10	1.13 (1.07-1.18)	0.29	0.79 (0.76-0.83)	0.63
	twin	12	1.11 (1-1.13)		0.79 (0.73-0.85)	
	triplet	21	1.08 (1.04-1.12)		0.77 (0.76-0.81)	

* critical value of Kruskal Wallis ANOVA rank test # scores obtained by dividing the number expressing child’s biological age by the number defining the standard age at which 50% of children population acquire a particular skill ## scores obtained by dividing the number expressing child’s biological age by the number defining the standard age at which 95% of children population acquire a particular skill

## Discussion

Few studies are currently available describing a correlation between multiple pregnancies and functional development of preterm children. The findings obtained from this study showed no statistically significant differences in functional development of premature children born as singletons, twins, or triplets between the 32nd and 36th week of pregnancy. Presented results are consistent with other national research into this topic. The study conducted by Bieleninik et al. also did not show any statistically significant differences between psychomotor development of preterm twins and singletons. The average age of the examined singletons was 32 months; twins had an average age of 34 months. The Bayley III scale, which was used to examine the children’s psychomotor development, included assessment of their cognition and psychomotor and language skills [3]. Chrzan-Dętkoś et al. also did not find statistically significant differences between preterm singletons and twins as far as their intellectual and motor development was concerned [12]. Similar conclusions could be found in some foreign studies. Kyriakidou et al. compared the development of preterm children born from singleton and multiple pregnancies between the 25th and 34th week by means of the Bayley III scale and Toddler Development III. After 24 months of observation, the researchers did not observe any statistically significant differences in cognitive and motor development between preterm singletons and twins [13]. A retrospective cohort population study conducted by Gnanendran et al. examined preterm children born from singleton and multiple pregnancies before the 29th gestational week. A neurodevelopmental assessment carried out with the application of Bayley scale II at the corrected age of two to three did not show statistically significant differences in moderate and severe disability between preterm children born from singleton pregnancies and those born from multiple pregnancies. Preterm children born from multiple and singleton pregnancies obtained comparable neurodevelopmental scores [14]. A population-based study, EPIPAGE, conducted in France in 1997 did not show significant differences between twins and singletons as far as the frequency of the incidence of cerebral palsy was concerned [15]. Raz et al. also found that no differences were apparent in the motor development index between preterm twins and preterm singletons. However, the scores obtained from language processing tasks were much lower in the case of twins as compared with preterm singletons [16]. In a retrospective cohort study conducted by Tabord and Guiomar, the development of preterm children born before the 32nd week of pregnancy and infants born with very low birth weight was assessed at the age of 24 months with the application of the Growing Skills II Scale. The findings of this study showed a higher index of moderate and severe neurodevelopmental disorders in twins in comparison with children born from singleton pregnancies [17].

All in all, a vast majority of the available research leads to conclusions similar to the ones drawn from this study. This study’s novel approach to the problem of psychomotor development of preterm babies born from multiple pregnancies consists in examining children without serious complications caused by premature birth, such as neurological disorders or sight and hearing disorders, which might affect the outcome of functional development diagnosis. The study is also innovative because of the application of Munich Functional Developmental Diagnostics, which makes it possible to assess functional development in seven separate spheres, such as walking age, manual skilfulness, scope of perception, speech development, speech understanding, social age and the scope of independence.

The values of quotient medians for the 50th percentile in all spheres of functional development fluctuate around 1, which allows the conclusion that moderate and late preterm children do not differ significantly from the general population of children aged between 2 and 2.5. Scientific publications suggest that late and moderate preterm children are at higher risk of developmental disorders in early childhood in comparison with children born from full-term pregnancies, but it is also pointed out that some preterm children quickly catch up with gross and fine motor skills, speaking skills, or cognitive functions [18]. The lowest values of quotients were observed within manual skillfulness and walking age, which means that the examined children’s development for these skills is the most similar to the standard of the general population. Within the first two years of preterm children’s lives, gross motor skills are mainly monitored during medical check-ups and physiotherapist consultations. The first year of life is the most dynamic in the context of acquiring motor skills. Parents naturally put in a lot of effort to improve and control this aspect of their children’s development. The first step toward improving manual skillfulness of preterm children might be doing a puzzle or playing with building blocks or other manipulative toys adjusted to the children’s age in the company of their parents or babysitters. Preterm children’s parents usually try to arrange creative play for them to help them catch up with their peers born at full term. This might be the reason why the examined children scored the highest in this sphere of functional development.

Late preterm births are connected with a slight deficit of cognitive functions, which can frequently be observed at the age of 2. These problems might not be big enough to have clinical significance, but they might have a tendency to get worse. Cognitive development disorders in late preterm children are recorded at a later age, which leads to a conclusion that early tests and intervention might improve the cognitive development of this group of children [19,20]. Therefore, even though at the age of 2 to 2.5 the examined preterm children born between the 32nd and 36th week of pregnancy do not difer significantly from their peers born at full time, it does not mean that they do not need to be monitored during their succeeding childhood years.

In our opinion, the findings of the study indicate the necessity to plan further research projects into the functional development of preterm children born from singleton and multiple pregnancies.

### Study limitations

Project limitations include that the group of examined children was relatively small, and the number of children in particular groups differed. Moreover, the groups of twins and triplets consisted of children born of the same pregnancies. In research on the psychomotor development of premature infants, the use of the Bayley scale dominates in the world literature. In this study, the Munich Functional Developmental Diagnostics scale, which is not validated in Poland, was used.

## Conclusions

1. On the basis of the study group, no relationship was found between the multiplicity of pregnancies and the functional development of premature babies born between 32nd and 36th weeks of gestation.
2. At the age of 2 to 2.5, moderate and late preterm children without neurological disorders do not differ significantly from the general population of children born at full time in the spheres of walking skills, fine motor skills, perception, speech development and understanding, social age and the scope of independence.
3. Even though at the age of 2 to 2.5 the examined preterm children born between the 32nd and 36th week of pregnancy do not difer significantly from their peers born at full term, it does not mean that they do not need to be monitored during their succeeding childhood years.
4. It is necessary to further evaluate the functional development of the studied children at least until they reach school age.
5. It is necessary to conduct further research on a larger research sample.
